# Mechanistic insights into the anticancer effects of *Pinellia ternata* (Thunb.) Ten. ex Breitenb. and *Ligusticum chuanxiong* Hort. ex S. H. Qiu & al. on papillary thyroid carcinoma: A network pharmacology approach

**DOI:** 10.1097/MD.0000000000041841

**Published:** 2025-03-21

**Authors:** Gang Wang, Jing Chen, Xiangding Kong, Kuanyu Wang

**Affiliations:** a The First Affiliated Hospital of Heilongjiang University of Chinese Medicine, Harbin, Heilongjiang, China.

**Keywords:** *Ligusticum chuanxiong* Hort. ex S. H. Qiu & al, molecular docking, network pharmacology, papillary thyroid carcinoma, *Pinellia ternata* (Thunb.) Ten. ex Breitenb

## Abstract

This study aims to elucidate the mechanisms of action of *Pinellia ternata* (Thunb.) Ten. ex Breitenb. and *Ligusticum chuanxiong* Hort. ex S. H. Qiu & al. (PAL) in treating papillary thyroid carcinoma (PTC) using bioinformatics and network analysis. Compounds in PAL were identified from the HERB database. Potential herbal targets were predicted using the SwissADME and SwissTargetPrediction platforms. Differential expression genes related to PTC were extracted from the GEO database and protein–protein interaction networks were constructed using the String database and Cytoscape software. Additionally, gene ontology and Kyoto Encyclopedia of Genes and Genomes enrichment analyses were conducted, and core compound-target interactions were validated through molecular docking. Effective components identified included 32 from *Pinellia ternata* (Thunb.) Ten. ex Breitenb. and 105 from *Ligusticum chuanxiong* Hort. ex S. H. Qiu & al., comprising 825 targets. A total of 2155 differential expression genes related to PTC were selected using GEO2R software, with 71 therapeutic targets identified. Gene ontology and Kyoto Encyclopedia of Genes and Genomes analyses suggest that PAL may exert effects through cancer-related pathways and signal transduction processes. Molecular docking indicated high binding affinity between several compounds and their targets. Specific active components in PAL may act through various mechanisms on PTC, offering scientific bases for further drug development and treatment strategies.

## 1. Introduction

Thyroid cancer, primarily papillary thyroid carcinoma (PTC), accounts for approximately 85% of all thyroid cancer cases and is one of the fastest-growing cancer incidences globally.^[1]^ Advances in medical diagnostics have increased the detection rates of PTC, particularly among women. The 10-year survival rate for PTC patients is about 95%, indicating a favorable prognosis, although the recurrence rate post-surgery can reach 20%.^[2,3]^ Current treatments for PTC include surgery, radioactive iodine therapy, and thyroid hormone suppression.^[4]^ However, treatment options remain limited for patients with recurrent or metastatic PTC, and the associated side effects cannot be overlooked.^[5]^

Traditional Chinese medicine (TCM) has gained international attention for its unique advantages and lower side effects in cancer treatment.^[6]^ *Pinellia ternata* (Thunb.) Ten. ex Breitenb. and *Ligusticum chuanxiong* Hort. ex S. H. Qiu & al., commonly used in TCM for treating scrofula and neck tumors, have shown inhibitory effects on various cancer cells.^[7,8]^ While *Pinellia ternata* (Thunb.) Ten. ex Breitenb. primarily modulates immune responses and suppresses tumor cell growth,^[9]^ *Ligusticum chuanxiong* Hort. ex S. H. Qiu & al. affects the tumor microenvironment, inhibits angiogenesis, and promotes cancer cell apoptosis.^[10]^ Despite preliminary studies revealing their anticancer potential, the specific mechanisms of their synergistic actions, particularly how they regulate multiple signaling pathways in PTC, remain unclear.

This study aims to explore the mechanisms of PAL in PTC treatment using a network pharmacology approach, which connects drugs, diseases, and targets through network models, reflecting the holistic and complex nature of drug actions in line with the holistic view and syndrome differentiation principles of TCM.^[11]^ By constructing a drug component-target network and integrating the pathological characteristics of PTC, this study systematically analyses the synergistic anticancer mechanisms of PAL, providing new insights and methods for treating PTC.

## 2. Materials and methods

### 2.1. Selection and target prediction of PAL active components

Utilising the HERB database, compounds within PAL were identified, integrating various TCM databases such as SymMap, TCMID, TCMSP, and TCMD, and analyzed using high-throughput sequencing. The SwissADME platform was employed to filter compounds following Lipinski’s rule, focusing on molecular weight < 500, ≤10 rotatable bonds, ≤10 H-bond acceptors, ≤5 H-bond donors, and a Log *P* value of ≤ 5. Pharmacokinetics parameters, such as high gastrointestinal absorption and blood–brain barrier permeability, were also considered, along with drug-likeness, requiring at least 3 “Yes” criteria. Potential targets for these compounds were predicted on the SwissTargetPrediction platform, selecting targets with a “Probability” >0.

### 2.2. Selection of PTC differential miRNA and related targets

The GEO database was searched using “Papillary thyroid carcinoma” as a keyword to find relevant chip datasets, including GSE3678, GSE33630, and GSE53157. Using GEO2R, these sample sets were analyzed to select differential expression genes associated with PTC under conditions of *P* < .05 and |log2FC|≥1.

### 2.3. Target screening and network construction for PAL treatment of PTC

PAL targets were merged with PTC targets to form an intersection set. The String database was used to construct a protein–protein interaction (PPI) network for the intersection targets, selecting those with a threshold >0.900. Cytoscape 3.8.0 was employed to create a “herbal-active component-therapeutic target-disease” network graph, selecting the top 5 compounds by Degree value as core compounds, which play a significant role in the PAL treatment process for PTC. Subsequently, PPIs were imported into Cytoscape for network topology analysis, selecting the top 5 targets by node degree as core targets.

### 2.4. Gene ontology enrichment analysis and Kyoto Encyclopedia of Genes and Genomes (KEGG) pathway enrichment analysis

Therapeutic targets were imported into the DAVID database with settings configured for Homo sapiens. Enrichment analyses were conducted under the condition of *P* < .05. The results, covering Biological Processes, Molecular Functions, and Cellular Components, were displayed using bubble charts. Using the BioMart platform, gene IDs of the therapeutic targets were converted to Ensemble gene IDs. The core targets underwent KEGG pathway enrichment analysis on the Omicshare platform, selecting entries with a *P* < .001, and the top 30 entries were visualized as a circular diagram.

### 2.5. Molecular docking validation

We performed targeted docking on DockThor using protein crystal structures (resolution < 2.5 Å) retrieved from the PDB. Water molecules and extraneous hetero atoms were removed with PyMol, and the protein was saved in a suitable format. For each protein, the docking box was centered on reported active sites or co-crystallized ligand positions, typically covering a 15 to 20 Å range. Default DockThor parameters were applied for grid size, scoring functions, and genetic algorithm settings. Each compound was docked ten times, and the top-ranked pose was chosen for analysis. Resulting complexes were inspected in PyMol to identify hydrogen bonds, hydrophobic contacts, and other interactions.

## 3. Results

### 3.1. Acquisition of herbal active components and targets

A total of 180 active components from *Pinellia ternata* (Thunb.) Ten. ex Breitenb. and 349 from *Ligusticum chuanxiong* Hort. ex S. H. Qiu & al. were retrieved from the HERB database. After filtering, 32 active components from *Pinellia ternata* (Thunb.) Ten. ex Breitenb. and 105 from *Ligusticum chuanxiong* Hort. ex S. H. Qiu & al. were selected (Tables S1 and S2, Supplemental Digital Content, http://links.lww.com/MD/O564, respectively). Using the SwissTargetPrediction platform, 5861 targets were initially predicted. After removing entries with a “Probability” of zero and duplicates, 825 targets remained.

### 3.2. Acquisition of PTC differential expression genes

**Figure 1.**
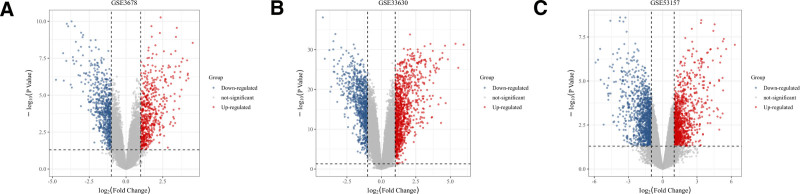
(A) Shows the volcano plot of differentially expressed genes for the dataset GSE3678, with 556 genes upregulated and 635 genes downregulated. (B) Shows the volcano plot of differentially expressed genes for the dataset GSE33630, with 1067 genes upregulated and 841 genes downregulated. (C) Shows the volcano plot of differentially expressed genes for the dataset GSE53157, with 1029 genes upregulated and 1187 genes downregulated.

The GEO dataset GSE3678 contained 14 samples, with 7 in the PTC group and 7 in the control group (Fig. 1A). The GSE33630 dataset contained 105 samples, with 49 in the PTC group, 45 in the control group, and 11 samples of undifferentiated thyroid cancer (Fig. 1B). The GSE53157 dataset included 26 samples, with 7 in the PTC group, 2 in the control group, and additional samples of less differentiated cancers (Fig. 1C). Differential gene expression profiles were obtained through microarray analysis. Using a criterion of *P* < .05 and |log2FC| ≥1, 1531, 1243, and 869 differential expression genes were identified in datasets GSE3678, GSE33630, and GSE53157, respectively. After merging and removing duplicates, a total of 2155 relevant genes were identified.

### 3.3. Selection of therapeutic targets and network construction

**Figure 2.**
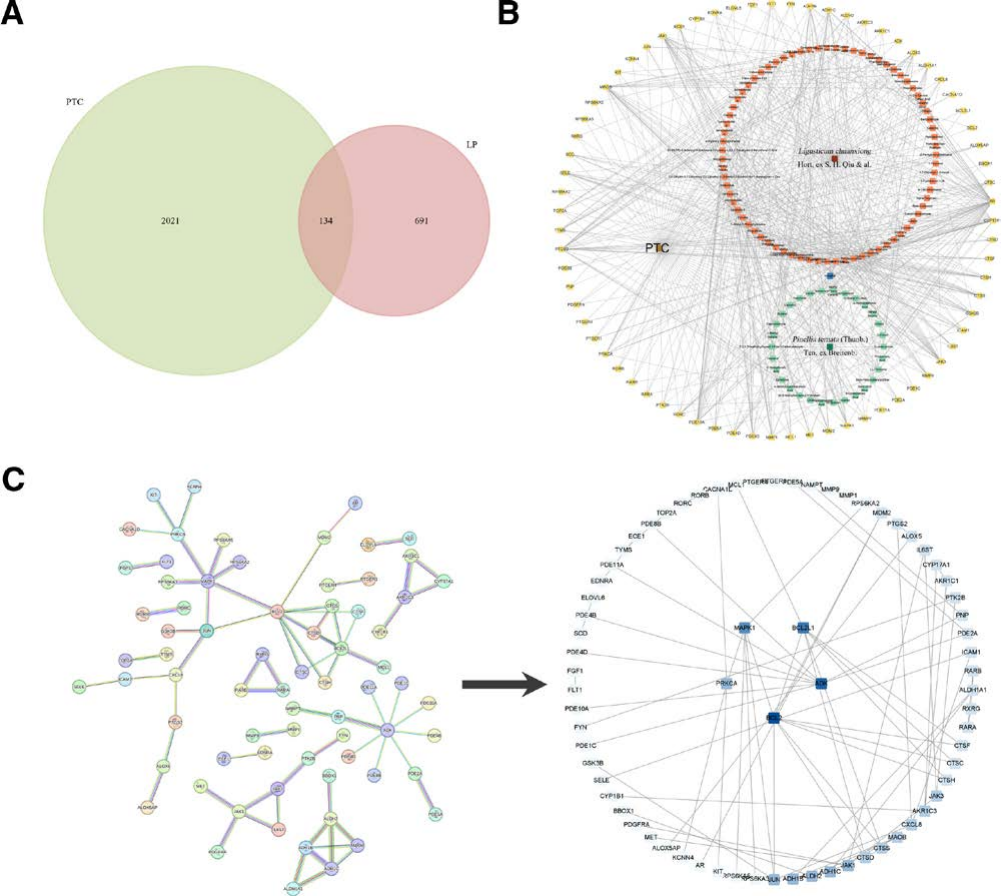
(A) Displays a Venn diagram of the intersection targets between PAL and PTC. (B) Is a network diagram of the “Herbal-Active Component-Therapeutic Target-Disease” network. (C) Shows the PPI and core target screening network graph, where a darker color indicates greater importance of the target within the network.

A total of 134 targets were identified at the intersection of PAL and PTC targets (Fig. 2A). The String database was used for PPI analysis of these intersecting targets, yielding 72 interactions across 71 therapeutic targets. Using Cytoscape software, a “herbal-active component-therapeutic target-disease” network was constructed (Fig. 2B) and analyzed topologically. The selection of core compounds was based on degree centrality analysis of the compound-target network (Fig. 2B). The top 5 compounds with the highest degree values were identified (Neocnidilide, 2-Undecanone, Citronellyl Acetate, Senkyunolide K, and Senkyunone). However, as Senkyunone, Levistolid A, and Neryl Acetate exhibited identical degree values, a total of 7 compounds were ultimately retained as core constituents. Additionally, through topological analysis of the PPI network, 5 core targets were selected: BCL2, ADK, BCL2L1, MAPK1, and PRKCA (Fig. 2C).

### 3.4. Enrichment analysis results

**Figure 3.**
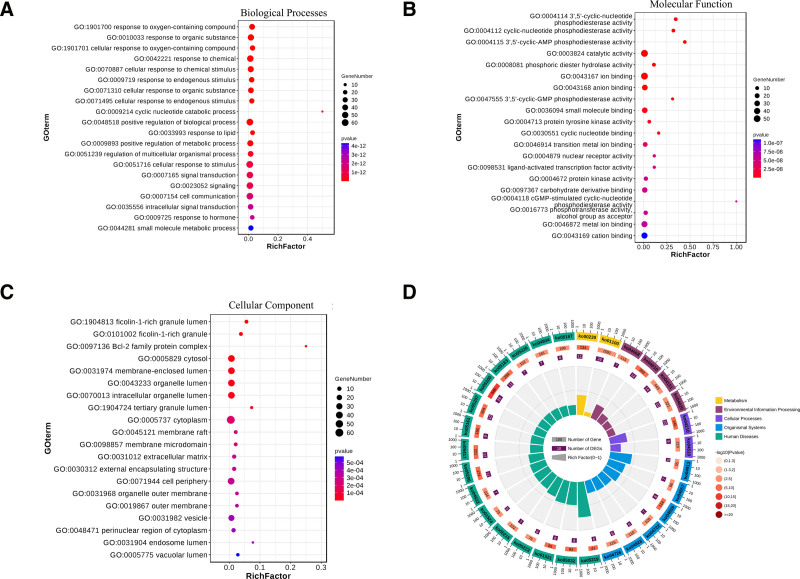
(A) Is a bubble chart for biological processes results. (B) Is a bubble chart for molecular functions results. (C) Is a bubble chart for cellular components results; in the bubble charts, colors transition from red to blue, indicating increasing *P*-values. (D) Shows the top 30 significant KEGG pathways, with bar length corresponding to the number of background genes, and the depth of color corresponding to the *P*-value, with darker colors indicating smaller values.

A total of 2270 entries were obtained from the gene ontology enrichment analysis under the selection criterion of *P* < .05, with Biological Processes accounting for 1872 entries, including responses to oxygen-containing compounds, organic substances, and chemicals (Fig. 3A). Molecular Functions included 288 entries, such as 3’,5’-cyclic-nucleotide phosphodiesterase activity, phosphoric diester hydrolase activity, and protein tyrosine kinase activity (Fig. 3B). Cellular Components comprised 110 entries, including the ficolin-1-rich granule lumen, Bcl-2 family protein complex, and cytosol (Fig. 3C). The KEGG pathway enrichment analysis resulted in 32 entries under the criterion of *P* < .001, with cancer-related pathways being most prevalent, accounting for 31.25% of entries, including Pathways in cancer, MicroRNAs in cancer, and Chemical carcinogenesis – receptor activation. Additional pathways enriched in PAL’s treatment of PTC included endocrine-related pathways, such as the AGE-RAGE signaling pathway in diabetic complications and Parathyroid hormone synthesis, secretion and action; immune-related pathways, such as Th17 cell differentiation and the IL-17 signaling pathway; and various signaling pathways, including the MAPK signaling pathway, PI3K-Akt signaling pathway, TNF signaling pathway, and JAK-STAT signaling pathway (Fig. 3D).

### 3.5. Molecular docking results

**Figure 4.**
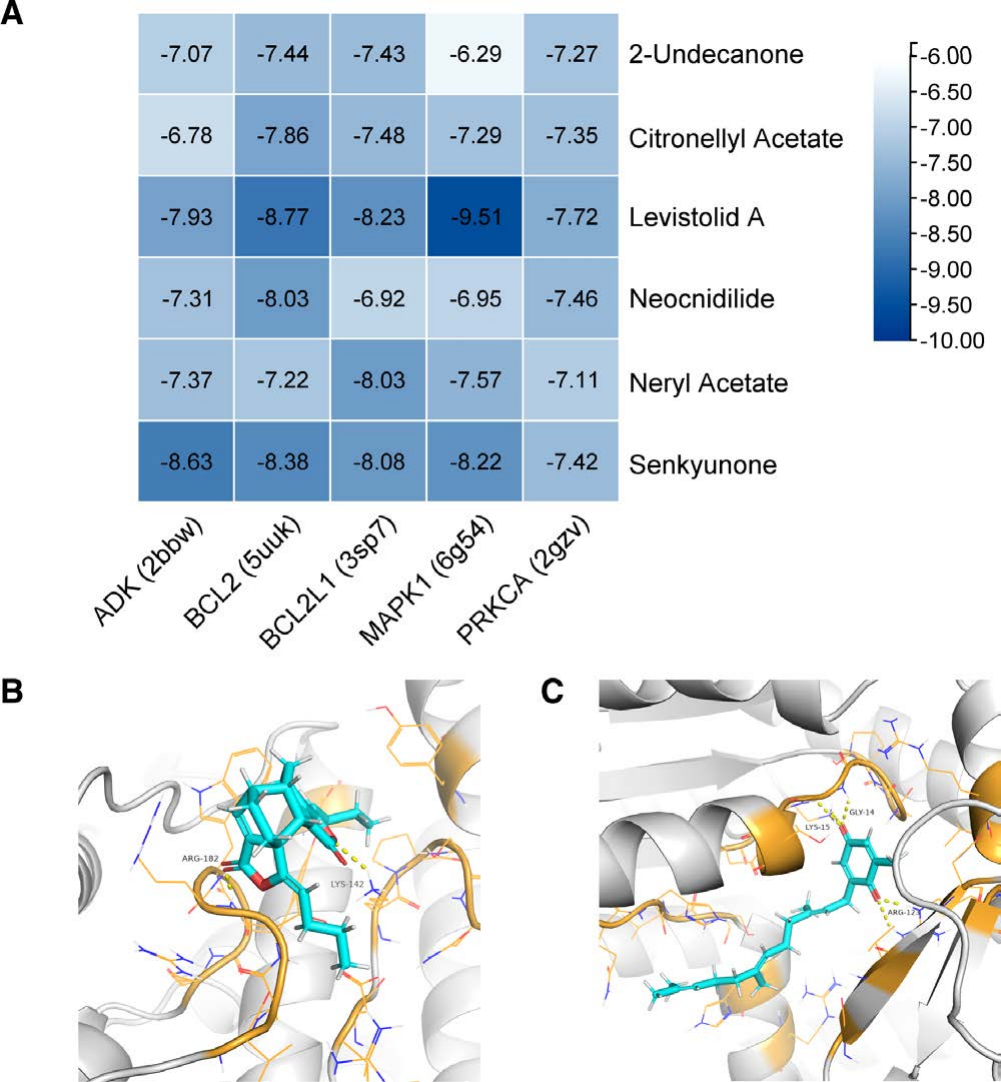
(A) Is a heatmap of the molecular docking results, with colors transitioning from yellow to purple as the docking scores decrease. (B) Is a schematic of the binding between Levistolide A and MAPK1. They form 1 hydrogen bond at Arginine 182 and 1 hydrogen bond at Lysine 142. (C) Is a schematic of the binding between Senkyunone and ADK. They form 2 hydrogen bonds at Arginine 123, 1 hydrogen bond at Lysine 15, and 2 hydrogen bonds at Glycine 14.

Core compounds and core targets selected in this study were subjected to molecular docking. Senkyunolide K was excluded as its 3D structure is not available in PubChem. The heatmap of docking results (Fig. 4A) showed all docking scores < −6.0 kcal/mol, with an average docking score of −7.64 kcal/mol, and 86.7% of the results had docking scores ≤ −7.0 kcal/mol, demonstrating good binding capabilities. The compounds with better binding capabilities were Levistolide A and Senkyunone, both with average docking scores < −8.0 kcal/mol. High-scoring docking results for these compounds were visualized using PyMol (Fig. 4B, C).

## 4. Discussion

PTC, known for its relatively good prognosis yet high recurrence rate, presents ongoing challenges in treatment, especially for cases involving recurrence or distant metastasis.^[12]^ This study employed network pharmacological approaches to explore the potential therapeutic mechanisms of PAL in PTC, suggesting that PAL might exert anticancer effects by influencing multiple biological processes and signaling pathways.

Through high-throughput data analysis, this research identified 137 active compounds and 71 potential therapeutic targets, with 7 compounds demonstrating strong associations with key cancer targets. Notably, Levistolide A and Senkyunone not only occupy central positions within the network but also exhibit strong binding capabilities in molecular docking experiments, indicating their potential as robust therapeutic agents against PTC. Levistolide A, a butenolide, has shown significant effects in inhibiting cancer cell proliferation and inducing apoptosis. Studies indicate that Levistolide A directly targets cyclin-dependent kinases, thereby disrupting the progression of the cell cycle and causing cell cycle arrest at the G1/S or G2/M phases.^[13]^ This action decreases the frequency of cell division, slowing tumor growth. Furthermore, Levistolide A can induce the activation of the pro-apoptotic protein Bax and inhibit the expression of the antiapoptotic protein Bcl-2, mechanisms that promote programmed cell death in cancer cells.^[14]^ Regarding signaling pathways, Levistolide A inhibits the activation of MAPK/ERK and PI3K/Akt pathways, thus suppressing tumor cell survival and proliferation signals, enhancing the efficacy of chemotherapeutic drugs, and reducing tumor resistance to treatment.^[15]^ Senkyunone, a class of furanocoumarin organic compounds, has been researched for treating various cancers, including PTC. Its anticancer role is primarily demonstrated in its antiproliferative, antimetastatic, and apoptosis-inducing capabilities. Similar to Levistolide A, Senkyunone can also modulate Bcl-2 activity, halting the cell cycle and causing cancer cell growth arrest.^[16]^ Additionally, Senkyunone enhances the release of cytochrome c within the mitochondrial pathway and activates caspase family proteins, thereby inducing apoptosis in cancer cells.^[17]^ In terms of antimetastatic properties, Senkyunone inhibits the activity of extracellular matrix-degrading enzymes such as matrix metalloproteinases, reducing the migratory ability of cancer cells and preventing their spread through the blood or lymphatic systems.^[18]^ Thus, Levistolide A and Senkyunone, by regulating key cell cycle proteins and pro-apoptotic pathways, as well as inhibiting tumor cell migration and invasion, exhibit significant anticancer potential and offer new targets and strategies for treating PTC.

The therapeutic potential of distinct core compounds may be further amplified through synergistic interactions, leveraging their ability to target multiple critical signaling hubs in PTC, thereby offering the advantage of multi-target intervention. Network proximity analysis revealed that 85% of compound pairs act on proteins within 2 network steps, suggesting potential complementary mechanisms. The combination of Neocnidilide (a STAT3 inhibitor) and Citronellyl Acetate (an EGFR blocker) could simultaneously suppress JAK-STAT and MAPK signaling pathways through dual-target inhibition, thereby curbing both proliferation and metastasis in PTC cells.^[19]^ Senkyunolide and 2-Undecanone may synergistically induce apoptosis via CASP3 activation and BCL2 inhibition, consistent with their overlapping targets in the apoptosis subnetwork.^[17,20]^ This polypharmacological synergy aligns with the principle of herb-pair compatibility in TCM.^[21]^

The KEGG enrichment analysis identified cancer-related pathways as the most significant in this study. The “Pathways in Cancer” encompasses a broad network involving key signaling pathways that regulate the cell cycle, survival, metabolism, and apoptosis, including PI3K/Akt, MAPK, and JAK-STAT. These pathways play a central role in the development and treatment of PTC. The PI3K/Akt signaling pathway, in particular, is one of the most studied pathways in PTC, promoting cell survival and proliferation while inhibiting apoptosis.^[22]^ In PTC, mutations or upregulation of the PIK3CA gene are common in cases with high invasiveness and treatment resistance.^[23]^ Additionally, the loss of function of PTEN, a negative regulator of this pathway, can lead to sustained Akt activation, promoting tumor progression.^[24]^ The MAPK pathway also holds a central place in PTC, especially the V600E mutation of the BRAF gene, the most common genetic alteration in PTC, leading to abnormal activation of the MAPK pathway, enhancing cell proliferation, and antiapoptotic capabilities.^[25]^ Currently, targeted therapies against the BRAF mutation are a crucial component of PTC treatment.^[26]^ The JAK-STAT pathway in PTC primarily influences tumor growth by mediating inflammatory responses and regulating the immune environment. Persistent activation of STAT3 in PTC is associated with poorer clinical outcomes.^[27]^ Studies have demonstrated that Senkyunone inhibits AKT1 phosphorylation while downregulating STAT3 activity. This suggests that STAT3 inhibitors may enhance radioactive iodine uptake in refractory PTC by reversing immunosuppressive microenvironments.^[28]^ Notably, the IL-6/JAK/STAT3 axis and PI3K-AKT pathway synergistically interact in PTC, forming a positive feedback loop that sustains tumor survival under hypoxic conditions.^[29]^ Such signaling crosstalk may explain the dual inhibitory effects of the identified compounds on both pathways. Additionally, cell cycle regulation is a critical component of the “Pathways in Cancer.” Disruptions in the expression of cell cycle regulators such as cyclins and CDK inhibitors like p21 and p27 are commonly observed in PTC, aiding cells in evading normal proliferation controls, leading to unrestrained cell division and tumor formation.^[30]^ These pathways are key signaling routes through which PAL might inhibit the proliferation and promote apoptosis of thyroid papillary carcinoma, thus manifesting its therapeutic effects.

Our network pharmacology and docking results highlight Levistolide A and Senkyunone, yet their ADME properties remain pivotal for actual therapeutic impact. While SwissADME screening suggests possible oral absorption, moderate solubility and first-pass metabolism could limit bioavailability. Techniques such as nano-formulations or prodrug modifications may enhance stability and target-tissue delivery, ensuring sufficient concentrations at the tumor site.^[31]^ Validating these in vitro and in vivo will be crucial to bridge computational insights and clinical efficacy.

Beyond mechanistic insight, combining PAL constituents with existing treatments for PTC may offer synergistic benefits. For instance, Levistolide A could be paired with BRAF or PI3K inhibitors to enhance tumor suppression, while Senkyunone’s pro-apoptotic effects may reduce chemo or radiotherapy doses. Standardizing extracts, conducting pharmacodynamic and pharmacokinetic studies, and identifying biomarker-driven subgroups (e.g., BRAF^V600E mutation) can help personalize therapy. Ultimately, integrating traditional usage with modern pharmacology will expedite the clinical application of PAL-based strategies, improving outcomes in PTC and possibly other malignancies.

Despite promising results, this study has limitations. Network and enrichment analyses have provided potential mechanisms and targets, which need further validation through in vivo and in vitro experiments. Moreover, the bioavailability and metabolic stability of TCM components also require further study to assess their practical efficacy and safety in clinical applications.

In conclusion, this research preliminarily reveals the potential mechanisms of PAL in the treatment of thyroid papillary carcinoma, providing a scientific basis for the future application of these TCM components in cancer therapy. Future studies could delve deeper into the mechanisms of these compounds and explore how to integrate these components more effectively into existing treatment protocols.

## 5. Related websites and software

BioMart platform provides a framework for gene ID conversion and is accessible at http://www.ensembl.org/biomart/martview. Cytoscape 3.8.0 is a software for network visualization and analysis, available at http://www.cytoscape.org/. DAVID database offers tools for gene functional classification and is available at https://david.ncifcrf.gov/. DockThor platform is used for molecular docking and can be found at https://dockthor.lncc.br/v2/. GEO database is a public repository for gene expression datasets, accessible at https://www.ncbi.nlm.nih.gov/geo/. HERB database integrates multiple TCM databases and is available at http://herb.ac.cn/. Omicshare platform facilitates pathway enrichment analysis and is available at http://www.omicshare.com/tools. PDB database hosts protein data and is accessible at https://www.rcsb.org/. PubChem database provides information about chemical substances, including their 3D structures, available at https://pubchem.ncbi.nlm.nih.gov/. PyMol 2.4.0 is a molecular visualization system, available at https://pymol.org/2/. String database offers PPI analysis, available at https://string-db.org/. SwissADME platform is used for screening compounds based on pharmacokinetic properties, available at http://www.swissadme.ch/. SwissTargetPrediction platform predicts potential drug targets and can be found at http://www.swisstargetprediction.ch/.

## Author contributions

**Conceptualization:** Jing Chen, Xiangding Kong.

**Writing – original draft:** Gang Wang, Kuanyu Wang.

**Writing – review & editing:** Kuanyu Wang.

## Supplementary Material

SUPPLEMENTARY MATERIAL
