# Supplementary material for: Mechanistic insights into the anticancer effects of Pinellia ternata (Thunb.) Ten. ex Breitenb. and Ligusticum chuanxiong Hort. ex S. H. Qiu & al. on papillary thyroid carcinoma: A network pharmacology approach
Source: Medicine (Baltimore). 2025 Mar 21;104(12):e41841. doi: 10.1097/MD.0000000000041841 (PMC11936596; doi:10.1097/MD.0000000000041841)
Supplement: SUPPLEMENTARY MATERIAL [file medi-104-e41841-s001.pdf]

Table S1. Active ingredients of *Pinellia ternata* (Thunb.) Ten. ex Breitenb.

Component	Formula	Molecular Weight	Rotatable Bonds	H-Bond Acceptors	H-Bond Donors	Consensus Log P	Gi Absorption	Bbb Permeant	Lipinski	Ghose	Veber	Egan	Muegge
(-)-Citronellal	C10H18O	154.3	5	1	0	2.94	High	Yes	Yes	No	Yes	Yes	No
2-Undecanone	C11H22O	170.3	8	1	0	3.48	High	Yes	Yes	Yes	Yes	Yes	No
3-Phenylpropionic Acid	C9H10O2	150.2	3	2	1	1.78	High	Yes	Yes	No	Yes	Yes	No
4-Methoxycyclohexanoic acid	C8H8O3	152.2	2	3	1	1.49	High	Yes	Yes	No	Yes	Yes	No
6-Shogaol	C17H24O3	276.4	9	3	1	3.76	High	Yes	Yes	Yes	Yes	Yes	Yes
9-Oxononanoic Acid	C9H16O3	172.2	8	3	1	1.71	High	Yes	Yes	Yes	Yes	Yes	No
Anethole	C10H12O	148.2	2	1	0	2.79	High	Yes	Yes	No	Yes	Yes	No
Benzaldehyde	C7H6O	106.1	1	1	0	1.57	High	Yes	Yes	No	Yes	Yes	No
Bis(4-Hydroxybenzyl)Ether	C14H14O3	230.3	4	3	2	2.34	High	Yes	Yes	Yes	Yes	Yes	Yes
Butyl vinyl ether	C6H12O	100.2	4	1	0	1.82	High	Yes	Yes	No	Yes	Yes	No
Catechol	C6H6O2	110.1	0	2	2	0.97	High	Yes	Yes	No	Yes	Yes	No
Cavidine	C21H23NO4	353.4	2	5	0	3.2	High	Yes	Yes	Yes	Yes	Yes	Yes
Cedrol	C15H26O	222.4	0	1	1	3.55	High	Yes	Yes	Yes	Yes	Yes	No
Citral	C10H16O	152.2	4	1	0	2.71	High	Yes	Yes	No	Yes	Yes	No
Coniine	C8H17N	127.2	2	1	1	1.99	High	Yes	Yes	No	Yes	Yes	No
Conimine	C22H36N2	328.5	1	2	2	3.84	High	Yes	No	Yes	Yes	Yes	Yes
Crysophanol	C15H10O4	254.2	0	4	2	2.38	High	Yes	Yes	Yes	Yes	Yes	Yes
Ephedrine	C10H15NO	165.2	3	2	2	1.46	High	Yes	Yes	Yes	Yes	Yes	No

Ferulic Acid	C10H10O4	194.2	3	4	2	1.36	High	Yes	Yes	Yes	Yes	Yes	No
Furfural	C5H4O2	96.08	1	2	0	0.69	High	Yes	Yes	No	Yes	Yes	No
Hydroquinone	C6H6O2	110.1	0	2	2	0.87	High	Yes	Yes	No	Yes	Yes	No
Methyl 2-Chloropropenoate	C4H5ClO2	120.5	2	2	0	1.17	High	Yes	Yes	No	Yes	Yes	No
Methylpyrazine	C5H6N2	94.11	0	2	0	0.62	High	Yes	Yes	No	Yes	Yes	No
N-(5-Methylisoxazol-3-Yl)Acetamide	C6H8N2O2	140.1	2	3	1	0.61	High	Yes	Yes	No	Yes	Yes	No
Nonanal	C9H18O	142.2	7	1	0	2.78	High	Yes	Yes	No	Yes	Yes	No
Norharman	C11H8N2	168.2	0	1	1	2.41	High	Yes	Yes	Yes	Yes	Yes	No
O-Methylferulic Acid	C11H12O4	208.2	4	4	1	1.83	High	Yes	Yes	Yes	Yes	Yes	Yes
P-Coumaric Acid	C9H8O3	164.2	2	3	2	1.26	High	Yes	Yes	Yes	Yes	Yes	No
Protocatechuic Aldehyde	C7H6O3	138.1	1	3	2	0.8	High	Yes	Yes	No	Yes	Yes	No
Scopoletin	C10H8O4	192.2	1	4	1	1.52	High	Yes	Yes	Yes	Yes	Yes	No
Spantol	C10H13NO2	179.2	5	2	1	1.8	High	Yes	Yes	Yes	Yes	Yes	No
Valeraldoxime	C5H11NO	101.2	3	2	1	1.32	High	Yes	Yes	No	Yes	Yes	No

Table S2. Active ingredients of *Ligusticum chuanxiong* Hort. ex S. H. Qiu & al.

Chemical Compound	Canonical SMILES	Formula	MW	#Rotatable bonds	#H-bond acceptors	#H-bond donors	Consensus Log P	GI absorption	BBB permeant	Lipinski #violations	Ghose #violations	Veber #violations	Egan #violations
(+)-Cis-Carveol	CC(=C)C1CC=C(C(C1)O)C	C10H16O	152.23	1	1	1	2.43	High	Yes	Yes	No	Yes	Yes
(2-Pentylphenyl)Methanol	CCCCCC1CCCCC1CO	C12H18O	178.27	5	1	1	3.13	High	Yes	Yes	Yes	Yes	Yes
(S)-2,2,3-Trimethylcyclopent-3-Ene-1-Acetaldehyde	O=CCC1CC=C(C1(C)C)C	C10H16O	152.23	2	1	0	2.29	High	Yes	Yes	No	Yes	Yes
1,1-Diethoxybutane	CCCC(OCC)OCC	C8H18O2	146.23	6	2	0	2.1	High	Yes	Yes	No	Yes	Yes
1,3-Benzodioxole	C1OC2C(O1)CCCC2	C7H6O2	122.12	0	2	0	1.71	High	Yes	Yes	No	Yes	Yes
1,4-Cineole	CC(C12CCC(O2)(CC1)C)C	C10H18O	154.25	1	1	0	2.77	High	Yes	Yes	No	Yes	Yes
1-Acetyl-2-Phenylhydrazine	CC(=O)NNC1CCCCC1	C8H10N2O	150.18	3	1	2	1.05	High	Yes	Yes	No	Yes	Yes
1-Acetyl-Beta-Carboline	CC(=O)C1NCCC2C1[NH]C1C2CCCC1	C13H10N2O	210.23	1	2	1	2.38	High	Yes	Yes	Yes	Yes	Yes

1-Methoxy-2-Methylanthraquinone	COC1C(C)CCC2C1C(=O)C1C(C2=O)CCCC1	C16H12O3	252.26	1	3	0	2.81	High	Yes	Yes	Yes	Yes	Yes
1-Terpineol	CC(C1=CCC(CC1)(C)O)C	C10H18O	154.25	1	1	1	2.34	High	Yes	Yes	No	Yes	Yes
2,2,3-Trimethylcyclopent-3-Ene-1-Carboxaldehyde	O=CC1CC=C(C1(C)C)C	C9H14O	138.21	1	1	0	2.01	High	Yes	Yes	No	Yes	Yes
2,3-Dihydro-5,7-Dihydroxy-2,6-Dimethyl-8-(3-Methyl-2-Butenyl)-4H-1-Benzopyran-4-One	CC1CC(=O)C2C(O1)C(CC=C(C)C)C(C(C2O)C)O	C16H20O4	276.33	2	4	2	3.1	High	Yes	Yes	Yes	Yes	Yes
2,7-Dimethyl-1-Octanol	OCC(CCCCC(C)C)C	C10H22O	158.28	6	1	1	3	High	Yes	Yes	No	Yes	Yes
2-Methylbenzoxazole	CC1NC2C(O1)CCCC2	C8H7NO	133.15	0	2	0	2.06	High	Yes	Yes	No	Yes	Yes
2-Propionylfuran	CCC(=O)C1CCCO1	C7H8O2	124.14	2	2	0	1.37	High	Yes	Yes	No	Yes	Yes
3-Cyclohexen-1-Ol	OC1CCC=CC1	C6H10O	98.14	0	1	1	1.23	High	Yes	Yes	No	Yes	Yes

3-Methyl Butanal	O=CCC(C)C	C5H10O	86.13	2	1	0	1.14	High	Yes	Yes	No	Yes	Yes
3Z,6S,7R)-6-Butanoyl-3-Butylidene-7-Hydroxy-4,5,6,7-Tetrahydro-2-Benzofuran-1-One	CCCC=C1OC(=O)C2=C1CCC(C2O)C(=O)CCC	C16H22O4	278.34	5	4	1	2.54	High	Yes	Yes	Yes	Yes	Yes
4-Carvomenthenol	CC1=CCC(CC1)(O)C(C)C	C10H18O	154.25	1	1	1	2.6	High	Yes	Yes	No	Yes	Yes
4-Hydroxy-3-Butylphthalide	CCCCC1OC(=O)C2C1C(O)CCC2	C12H14O3	206.24	3	3	1	2.37	High	Yes	Yes	Yes	Yes	Yes
4-Hydroxybenzoic Acid	OC1CCC(CC1)C(=O)O	C7H6O3	138.12	1	3	2	1.05	High	Yes	Yes	No	Yes	Yes
4-Iodoindoline	IC1CCCC2C1CCN2	C8H8IN	245.06	0	0	1	2.41	High	Yes	Yes	No	Yes	Yes
4-Octanone	CCCCC(=O)CCC	C8H16O	128.21	5	1	0	2.33	High	Yes	Yes	No	Yes	Yes
Alpha-Terpineol	CC1=CCC(CC1)C(O)(C)C	C10H18O	154.25	1	1	1	2.58	High	Yes	Yes	No	Yes	Yes
Aromadendrene Oxide 2	CC1CCC2C1C1C(C1(C)C)CCC12OC1	C15H24O	220.35	0	1	0	3.54	High	Yes	Yes	Yes	Yes	Yes
Benzyl Alcohol	OCC1CCCCC1	C7H8O	108.14	1	1	1	1.41	High	Yes	Yes	No	Yes	Yes

Beta-Asarone	CC=CC1CC(OC)C(CC1OC)OC	C12H16O3	208.25	4	3	0	2.7	High	Yes	Yes	Yes	Yes	Yes
Beta-Eudesmol	C=C1CCCC2(C1CC(CC2)C(O)(C)C)C	C15H26O	222.37	1	1	1	3.61	High	Yes	Yes	Yes	Yes	Yes
Butanal	CCCC=O	C4H8O	72.11	2	1	0	0.9	High	Yes	Yes	No	Yes	Yes
Butylidene Phthalide	CCCC=C1OC(=O)C2C1CCCC2	C12H12O2	188.22	2	2	0	2.94	High	Yes	Yes	Yes	Yes	Yes
Butylphthalide	CCCCC1OC(=O)C2C1CCCC2	C12H14O2	190.24	3	2	0	2.81	High	Yes	Yes	Yes	Yes	Yes
Caffeic Acid Dimethyl Ether	COC1CC(C=CC(=O)O)CCC1OC	C11H12O4	208.21	4	4	1	1.83	High	Yes	Yes	Yes	Yes	Yes
